# The Unilateral Jumping Structures of the Spotted Lanternfly, *Lycorma delicatula* (Hemiptera: Fulgoridae): A Highly Functional and Integrated Unit

**DOI:** 10.3390/biomimetics10070444

**Published:** 2025-07-06

**Authors:** Xu Chen, Aiping Liang

**Affiliations:** 1 Tianjin Key Laboratory of Conservation and Utilization of Animal Diversity, College of Life Sciences, Tianjin Normal University, 393 Binshuixidao Road, Xiqing District, Tianjin 300387, China; xchen@tjnu.edu.cn; 2 Key Laboratory of Zoological Systematics and Evolution, Institute of Zoology, Chinese Academy of Sciences, 1 Beichen West Road, Chaoyang District, Beijing 100101, China

**Keywords:** biomechanics, exoskeleton, kinematics, locomotion, morphology

## Abstract

Previous research on the jumping structures of insects with strong leaping abilities mainly focused on overall jumping mechanisms. Our study reveals that the unilateral jumping structures (UJSs) of *L. delicatula* has relative functional autonomy. The UJSs consist of three distinct but interconnected parts: (1) energy storage component: it comprises the pleural arch and trochanteral depressor muscles, with the deformation zone extending about two-thirds of the pleural arch from the V-notch to the U-notch; (2) coupling component: made up of the coxa and trochanter, it serves as a bridge between the energy and lever components, connecting them via protuberances and pivots; and (3) lever component: it encompasses the femur, tibia, and tarsus. A complete jumping action lasts from 2.4 ms to 4.6 ms. During a jump, the deformation length of the pleural arch is 0.96 ± 0.06 mm. The angles ∠ct (angle between coxa and trochanter), ∠fp (angle between femur and pleural arch), and ∠ft (angle between femur and tibia) change by 57.42 ± 1.60, 101.40 ± 1.59, and 36.06 ± 2.41 degrees, respectively. In this study, we abstracted the jumping structures of *L. delicatula* and identified its critical components. The insights obtained from this study are anticipated to provide valuable inspiration for the design and fabrication of biomimetic jumping mechanisms.

## 1. Introduction

Jumping represents a crucial mode of locomotion employed by a diverse array of animal species [1]. Despite the varying habitats inhabited by different animals, the act of jumping is frequently triggered as a response to urgent threats and serves as a means of evading danger. Consequently, to execute a jump, animals must accumulate energy and then rapidly release it in times of emergency. The physical structures of animals vary greatly, resulting in a wide variety in the mechanisms utilized for jumping [2,3]. Moreover, jumping endows certain winged insects with the remarkable ability to achieve rapid take-off [4].

Arthropods often utilize the elastic properties of their exoskeleton as a means of storing energy for jumping [5]. Among arthropods, many Hemiptera insects exhibit exceptional jumping abilities. The jump of these insects is executed by metathoracic muscles, which are directly connected to the hind leg trochanter and responsible for the movements of both coxa (the first or basal segment of the leg of insect) and trochanter (the second segment of the leg of insect adjacent to the coxa) [6]. The pleural arches (it is a part of the internal skeleton of the thorax, which is bow-shaped, located on both sides of the metathorax, and it deforms during jumping to store and release energy) are bent by the contractions of the trochanteral depressor muscles (the muscles mainly consist of peripheral muscle fibers and a core part, and they contract to bend the pleural arch for energy storage, being crucial muscles for the storage and release of energy in jumping) [7]. These structures have been observed in many Hemiptera insects, which are prolific jumpers, such as Cercopoidea or froghoppers [8,9], Cicadelloidea or leafhoppers [10,11], Eurymelinae or treehoppers [12], and Fulgoroidea or planthoppers [2,13,14,15]. The jump ability of planthoppers (*Issus coleoptratus*) ranks alongside the froghopper (*Philaenus spumarius*), which is one of the best jumping insects [2,8,9]. While the deformation of the pleural arch has been examined in several froghoppers and planthoppers [5,7,16], the exact deformation characters of it during the jumping has yet to be explored.

The jumping structures of insects are intricately linked to their lifestyles and have undergone long-term evolution. The froghoppers, planthoppers, treehoppers, and leafhoppers, with their piercing-sucking mouthparts, obtain their nutrients by sucking xylem. To acquire sufficient nutrients, they often need to remain fixed in one spot for extended periods. Additionally, most of these insects are xylem-sap feeders [17] or partially xylem-sap feeders [18]. Due to the negative pressure and extremely low nutrient concentration in xylem fluid [19], xylem feeders must remain stationary for feeding most of the time. This makes them vulnerable to predators, necessitating an efficient and rapid means of escape when danger suddenly arises. Hence, the jump mechanisms ought to be streamlined and efficient, necessitating a level of autonomy and functional coherence within the jumping structures. This ensures seamless operation without the burden of intricate control procedures or undue dependence on additional bodily structures during the initiation of a jump.

Previous studies have primarily focused on the jumping mechanisms of these insects, treating the jumping structures as integral parts of the body [2,6,9,12,20]. Moreover, given that the jumping structures are symmetrically positioned on either side of the body, studies often emphasized coordinated bilateral movements [7,13,21,22]. For instance, dual-spring systems in trap-jaw ants facilitate ultrafast movements [23], and mantis shrimp appendages combine elasticity with leverage [24]. Notably, 3D modeling shows that spotted lanternfly nymphs exhibit simplified jumping kinematics compared to adults, emphasizing the importance of stage-specific analyses [25]. Furthermore, studies on the jumping mechanisms of froghoppers and leafhoppers have provided detailed anatomical and functional analyses of the hind leg structures, revealing the complex interplay between energy storage, muscle activation, and structural integrity [5,7,16,26].

Despite these advancements, there remains a gap in understanding the autonomous functionality of unilateral jumping structures in insects. This gap is particularly relevant in the context of biomimetic design, where the ability to isolate and replicate the functional units of biological systems can lead to innovative engineering solutions. Employing single-sided jumping structures as the focal point of research has the potential to effectively streamline the intricate biological architectures, thereby furnishing valuable insights and references for the fields of biomimetic design and manufacturing. This approach allows for a more focused and manageable exploration, facilitating the extraction of essential principles and features that can be translated into innovative engineering solutions. By isolating and studying these single-sided structures, we can better understand their unique characteristics and functional mechanisms, which in turn can inspire the development of more efficient and effective biomimetic products.

The spotted lanternfly, *Lycorma delicatula* (White) (Hemiptera: Fulgoridae), is a univoltine planthopper native to Asia that has recently spread to other countries [27,28,29]. It is highly polyphagous [30] and poses a threat to both agricultural and forest ecosystems [31]. In the nymphs’ stage, the *L. delicatula* already can jump [25,27], although the jumping ability is much weaker than that of an adult [31]. Adult *L. delicatula* has evolved multiple anti-predator defense strategies in morphology [32], chemistry [33], and behavior [28]. Among these strategies, jumping is the most direct and effective way to escape from danger. The jump behavior enables *L. delicatula* to move among host plants easily and disperse quickly [30]. The study of jumping in large lantern bugs showed that they were near isometrically scaled-up versions of their smaller relatives and still achieve comparable take-off velocities [34,35].

Previous study has demonstrated that dead planthopper *I. coleoptratus* can execute jumping movements through manual stimulation. However, it remains unclear whether this coordinated action relies exclusively on the hind legs or requires integration with other body segments, particularly regarding the mechanical interplay between specialized structures in the metathorax and the energy-storing components of the pleural arch [7].

The jumping performance of *L. delicatula* nymphs has been the focus of prior studies, which have primarily examined overall metrics (e.g., jump distance, take-off velocity) and general kinematic patterns [25]. However, key aspects of adult jumping, including the functional roles of their specialized jumping structures during propulsion, remain poorly characterized. To address this gap, we selected adult *L. delicatula* as the study subject, leveraging their robust jumping capability to investigate the biomechanics of jumping mechanisms in detail. Our preliminary investigations suggest that upon detachment from the insect, the unilateral jumping structures of *L. delicatula* retain the capacity to execute the jumping motion. This implies a certain degree of autonomy and functional integrity within the isolated structure.

In contrast to prior biomechanical investigations that conceptualized jumping structures as bilaterally integrated components dependent on whole-body coordination, our combined anatomical and kinematic analysis of unilateral jumping mechanisms in *L. delicatula* uncovers a self-contained mechanical architecture capable of autonomous operation without other body parts. By examining the structural–functional relationships within the unilateral jumping structures, we aim to bridge the translational gap between biological mechanisms and engineering applications through mechanistic characterization of component interactions. Elucidating the structural basis of independent locomotor function not only advances our understanding of biological motion but also establishes a theoretical framework for developing decentralized robotic systems and adaptive micro-mechanical devices capable of self-sustaining motion.

## 2. Materials and Methods

### 2.1. Animals

Adults of *L. delicatula* were collected on their host plants, *Ailanthus altissima*, in Beijing, China (116.387455° E, 40.009707° N), between July and September 2022. Based on our observation of the adults’ jumping behavior, it was found that females exhibited greater jump distance and height, larger body size, and larger muscle mass than males in preliminary trials. The enhanced jumping capabilities of females allowed us to capture more pronounced anatomical and kinetic changes during the jumping process. Consequently, females were selected as the subjects for this study. Based on our preliminary observations and knowledge from similar studies on insect biomechanics, we determined that a sample size of 10 females would be sufficient to capture the key morphological and kinetic characteristics of the unilateral jumping structures.

### 2.2. Anatomy

Observations on live insects were made on the same day of collection or after they had been in the laboratory for no more than 3 days feeding on live *A. altissima* plants in artificial climate chamber (MGC-HP, Shanghai Yiheng, Shanghai, China). Prior to the dissection process, females were put in plastic Petri dishes and transferred to a refrigerator maintained at 4 °C for a period of ten minutes, to diminish their movement and activity. Based on our initial observations and previous studies, a brief cooling treatment at 4 °C (typically lasting 10 min) does not cause significant adverse effects on tissue structural integrity or elasticity. Furthermore, live insects quickly regain full jumping ability following the 4 °C treatment, and the cold-induced temporary paralysis is completely reversible [36,37]. The unilateral jumping structures (UJSs), consisting of pleural arches and hind legs, were dissected from the females’ body in phosphate buffered saline (Solarbio Science & Technology, Beijing, China), and the anatomy of the hind legs and metathorax was examined with a Zeiss Discovery V12 stereo microscope (Oberkochen, Germany). Individual color photographs and videos were taken with a Nikon D7100 digital camera attached to the same microscope. Partially focused serial (30–50) images were combined in Helicon Focus software (Helicon Soft Ltd., Kharkiv, Ukraine) to produce completely focused images. The angles and lengths of structures were measured using ImageJ software (version 1.54d; National Institutes of Health, Maryland, USA). Measurements were taken using an ocular micrometer, and 10 females were measured. The data are presented as mean ± standard error (SE). The detailed explanations of the terminologies for anatomical structures are provided in the glossary within the Appendix A at the end of this article.

### 2.3. High-Speed Recordings of Jumping Actions

The unilateral jumping structures of the spotted lanternfly were carefully dissected under a Zeiss Discovery V12 stereo microscope and then placed in phosphate buffered solution. Then, the UJSs were fixed onto foam board with insect needles. The UJSs were manually loaded into its jumping-action-ready state by elevating the hind leg to the predetermined fixed position. In this latched state, the hind leg is firmly fixed in a specific position. Subsequently, the UJSs would maintain stability in this state for a certain period before spontaneously initiating the release. Jumping actions were recorded at a rate of 5000 frames per second using an i-SPEED 717 high-speed camera (iX Cameras, Rochford Essex, UK) equipped with a macro lens (SP 90 mm, Tamron, Saitama, Japan) from the ventral view. The start of the jumping motion was indicated by the rotation of the trochanter, which we designated as t = 0 ms. Following this, precise measurements were taken of the angles between the coxa and trochanter (∠ct), the femur and pleural arch (∠fp), and the femur and tibia (the fourth segment of the leg of insect, located between the femur and tarsus) (∠ft). Specifically, ∠ct was defined with the pivot as its vertex, one edge tracing the lower boundary of the coxa and the other aligning with the edge of the trochanter. For ∠fp, the vertex was placed at the juncture of the trochanter and femur, with one edge extending to the protrusion on the pleural arch from this juncture and the other following the femur’s centerline. The vertex for ∠ft was positioned at the juncture of the femur and tibia, with one edge running along the femur’s centerline and the other along the tibia’s centerline. The angles were chosen because they are critical for understanding the jumping mechanics of the UJS. These angles directly reflect the changes in the structure and position of the UJS components during the jumping process, providing insights into how energy is stored and released. Moreover, previous research on insect jumping mechanisms has shown that these angles are key indicators of the biomechanical performance of jumping structures, and the changes in these angles are closely related to the efficiency and direction control of jumping [2,7,9,26]. A total of 6 right and 4 left jumping units from female *L. delicatula* were recorded and measured.

Female ventral photographs were captured using a Nikon D7100 camera prior to jump initiation. Jump sequences were recorded at 5000 fps using the previously mentioned high-speed camera. Experiments were conducted in a rectangular chamber measuring 20 cm × 50 cm × 50 cm (W × H × D) with wooden flooring. The high-speed camera was positioned at the chamber’s front face, angled approximately 30° above horizontal to capture ventral abdominal and leg movements during jumping. Take-off was indicated when the hind legs lost contact with the ground. The acceleration time for take-off was defined as the period from the first detectable propulsive movement of the hind legs until take-off when the hind tarsi lost contact with the ground. Ten jumps of females were recorded.

The jumps and movements of specific leg joints were manually traced frame by frame using the Tracker software (https://physlets.org/tracker/, Version 6.3.0 accessed on 13 February 2025). To assess the potential influence of the force magnitude on the latched state, a comparative analysis was conducted between the UJS jumping—like motion and individual normal jump, focusing on the length of the pleural arch and the degrees of the leg points. The data are presented as mean ± standard error (SE). The differences in angles before and after jumping were assessed using a two-tailed unpaired *t*-test. All statistical analyses were conducted using SPSS Statistics Version 27.0 (IBM, New York, NY, USA).

## 3. Results

### 3.1. Morphology

**Figure 1:**
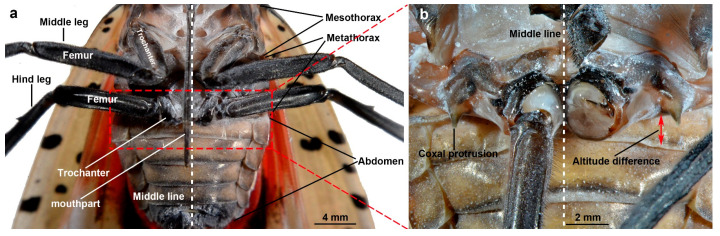
The position of jumping structures on the body of adult *L. delicatula*. (**a**) The ventral view of an adult *L. delicatula* from the mesothorax to abdomen. (**b**) The ventral view of the metathorax. The right hind leg was cut off to show the altitude difference between the metathorax and abdomen.

The jumping structures of adult *L. delicatula* are primarily located in the metathorax and hind legs. These structures are characterized by tough and dark fuscous skeletons. Among the various skeletal components, there exists some flexible, semitransparent membrane that acts as the connector (Figure 1). At the ventral junction between the metathorax and abdomen, specific sections of the metathorax and hind legs (coxae and trochanters) are positioned posterior to the abdomen, resulting in a notable altitude difference (Figure 1b).

#### 3.1.1. Jumping Components

**Figure 2:**
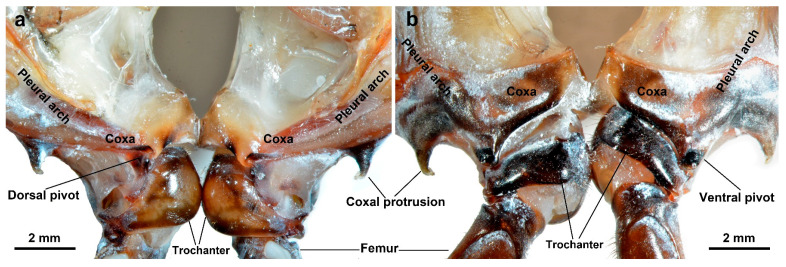
The bilateral jumping structures exist in pairs in the metathorax of an adult *L. delicatula*. (**a**) The dorsal view. (**b**) The ventral view.

The bilateral jumping structures of adult *L. delicatula* primarily consist of the pleural arches and hind legs (Figure 2). These structures are symmetrically arranged along the body. The pleural arches assume a bow-like shape and are situated on both sides of the metathorax. The coxae of the hind legs are lateral to the pleural arches on each side and are firmly fused to them. They align with each other and are securely fastened together at the ventral midline, enabling synchronized jumping movements. Furthermore, this connection enhances the overall stability of the body structure. Each coxal junction with the pleural arch features a small, pointed ventral protrusion.

**Figure 3:**
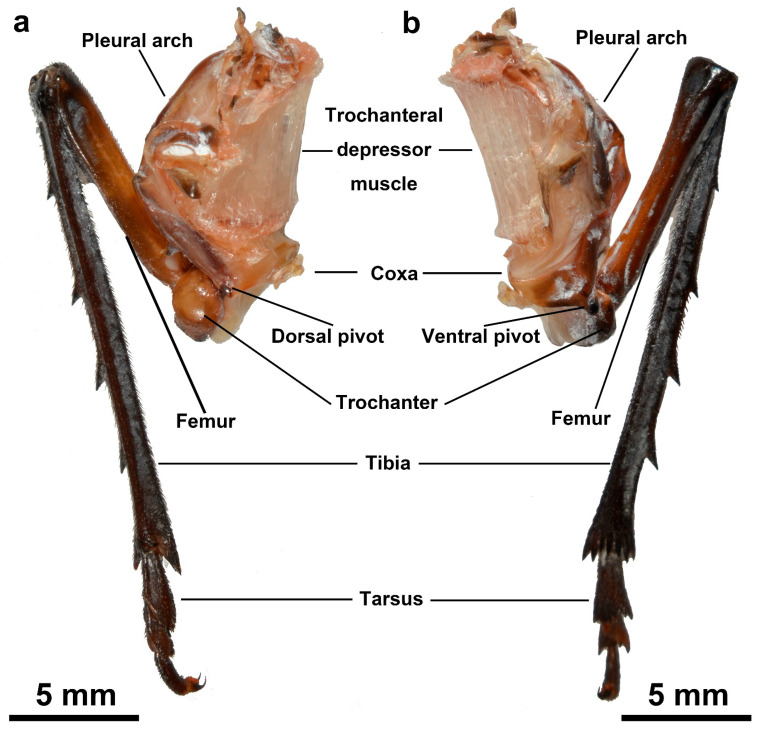
Detached unilateral jumping structures of adult *L. delicatula*. (**a**) Dorsal view. (**b**) Ventral view.

The unilateral jumping structures (UJSs) of *L. delicatula* comprises several distinct anatomical structures, including the pleural arch, the trochanteral depressor muscle, and the hind leg (composed of the coxa, trochanter, femur, tibia, and tarsus) (Figure 3). Each of these components serves distinct roles in the jumping mechanism, contributing to the coordinated biomechanical process that enables efficient locomotion. Despite existing in pairs and being connected to adjacent structures, the individual side of the jumping structures can operate independently when separated from the body. When the hind leg of the separated jumping mechanism is forcibly moved into its fully cocked position, it remains locked in place for a brief duration before automatically initiating the jumping action (supplementary Video S1). Video S1 showcases isolated unilateral jumping structures of *L. delicatula*. It demonstrates that even when detached from the body, the unit can perform the jumping motion, providing visual evidence of the relative autonomy of the unilateral jumping structure.

Based on their roles in the jumping mechanism, these components can be categorized as the energy storage component, the coupling component, and the lever component, respectively.

#### 3.1.2. Energy Store Component

**Figure 4:**
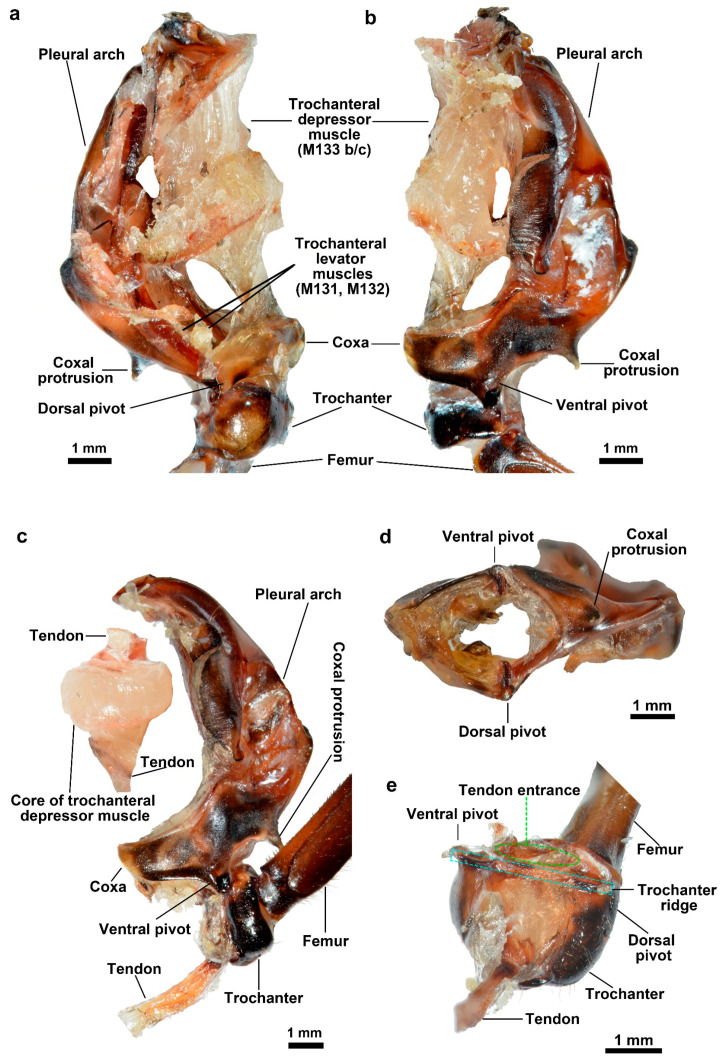
The energy store part and coupling part of a single jumping structure of adult *L. delicatula*. (**a**) The dorsal view, (**b**) ventral view, and (**c**) ventral view of the separated parts. The trochanteral depressor muscles were separated from the pleural arch to show the core of them and the tendon. (**d**) The antapical view of the coxa. There is a hole in the coxa for the tendon to pass through. (**e**) The separated trochanter.

The energy storage component consists of the pleural arch and the trochanteral depressor muscles, as illustrated in Figure 4a–c. The substantial trochanteral depressor muscles are primarily composed of two components: the peripheral muscle fibers and the core, which is surrounded and integrated by muscle fibers. After removing the surrounding muscle fibers, the core of the trochanteral depressor muscles become visible (Figure 4a,b). This core exhibits a translucent white color and a spindle-shaped structure. It is terminated by tendons at both ends and comprises disc-shaped connective tissue in the center (Figure 4c). The anterior tendon of the trochanteral depressor muscles inserts onto the anterior end of the pleural arch. Additionally, there are two posterior tendons of the trochanteral depressor muscles that insert onto the trochanter through the coxa hole (Figure 4d). One tendon inserts onto the side of the trochanter close to the midline (Figure 4c,e), while another tendon inserts onto the side of the trochanter away from the midline and enters through a hole of entry (Figure 4e). This tendon also connects with the femur and elevates it when preparing for a jump.

**Figure 5:**
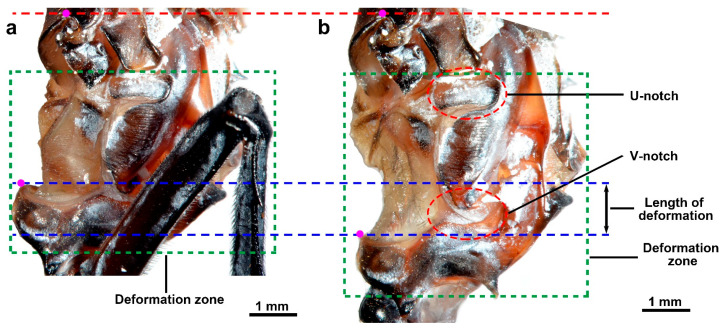
The deformation of the plural arch before and after jumping. (**a**) Before jumping. (**b**) After jumping. The red dotted line indicates the upper boundary of pleural arch; the blue dotted lines indicate the length of deformation. The green dotted line rectangles indicate the deformation zone. The pink dots indicate the endpoints at both ends when measuring the length of the plural arch.

Since the coxa is fused to the pleural arch, they are analyzed as a single unit when studying deformation. The pleural arch does not undergo uniform deformation during a jump, and it possesses two significant structures, the U-notch and V-notch, which are likely to play a significant role in its deformation during jumping. The deformation zone accounts for approximately two-thirds of the pleural arch, extending from the V-notch to the U-notch (Figure 5). Prior to releasing a jump, the pleural arch is bent by the trochanteral depressor muscles (Figure 5a). After jumping, the pleural arches recover their original shape, and the stored energy propels the coxa backward (Figure 5b).

#### 3.1.3. Coupling Component

The coupling component comprises the coxa and trochanter, collectively referred to as the coxa–trochanteral joint, which are the two segments of the hind leg at the base.

**Figure 6:**
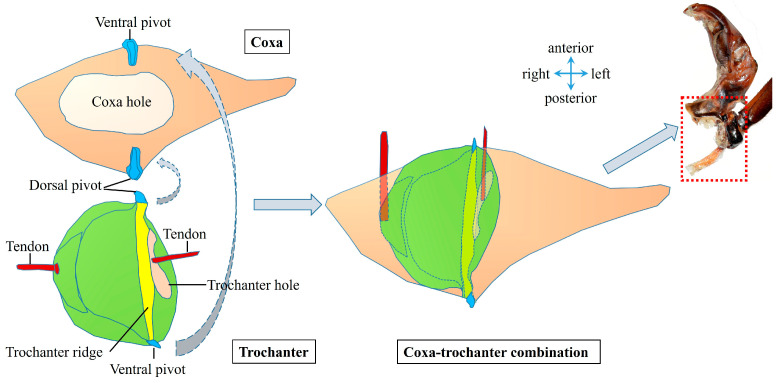
Schematic diagrams of the coupling between the coxa and trochanter.

The dorsal surface of the trochanter is hemispherical (Figure 4a), while the ventral surface is rectangular (Figure 4b). The main tendon, which links to the trochanteral depressor muscles, is inserted on the side close to the midline (Figure 4c,e; Figure 6) of the trochanter. On the other side, there is a hole for the tendon that connects to the trochanteral levator muscles to insert (Figure 4e; Figure 6). As shown in Figure 6, there are protuberances on both the ventral and dorsal sides, which couple with the ventral pivot and dorsal pivot on the coxa, respectively. Additionally, a ridge runs between them. Consequently, the trochanter assumes a seesaw-like structure. The pivots are situated on the coxa, and the two tendons on the opposite side of the trochanter generate tensile forces. The ridge serves as the spin axis. The main tendon and subordinate tendon pull the trochanter to rotate around the ridge (central axis) between the coupled pivots.

**Figure 7:**
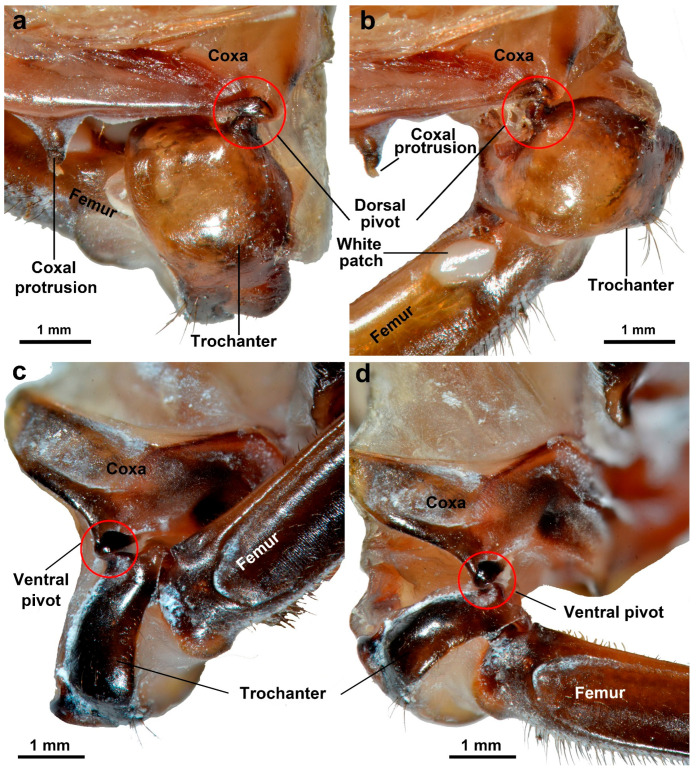
The position changes between the coxa and trochanter before and after jumping. (**a**) The dorsal view before jumping. (**b**) The dorsal view after jumping. (**c**) The ventral view before jumping. (**d**) The ventral view after jumping.

Furthermore, the protuberances on the trochanter coupled with the pivots on the coxa and their coupling play a crucial role in stabilizing the trochanter (Figure 7). Taking into account the observations obtained from this study as well as the existing relevant research, we hereby put forward the following proposed interactions between the coxa and the trochanter. The trochanter and coxa are locked together through the coupling of pivots, forming a combination. Subsequently, the contraction of the main trochanteral depressor muscles pulls the trochanter through the tendon they insert into. Since the trochanter and coxa are already locked together, the tensile force of the main trochanteral depressor muscles simultaneously pulls the coxa–trochanter combination. This leads to a bending of the pleural arch, effectively storing energy for jumping. The coxa–trochanter combination is maintained through a balance of tension from the trochanteral depressor muscles, trochanteral levator muscles, and the stabilization provided by the coupling of protuberances and pivots.

#### 3.1.4. Lever Component

The lever component is composed of the femur, tibia, and tarsus (the foot or fifth joint of the leg of the insect, typically consisting of several small segments and ending in a claw), which are the other three segments of the hind leg.

The length of the femur is 9.40 ± 0.07 mm (N = 10). It features a white cuticle patch that contrasts with the darker surrounding areas of the femoral cuticle. When a hind leg is fully elevated in preparation for jumping, the coxal protrusion engages with this specific patch of the femur (Figure 7). The length of the tibia is 19.36 ± 0.14 mm (N = 10). There are four spines on the tibia. The tibia is equipped with arc-shaped rows of distally oriented, strongly sclerotized spines, located ventrally on the distal margins. The length of the tarsus is 5.50 ± 0.06 mm (N = 10). The tarsus comprises two tarsal segments and a pretarsus. The pretarsus includes a pair of claws and an arolium positioned between them (Figure 3). The spines and claws serve to stabilize the body and prevent slipping during jumps.

**Figure 8:**
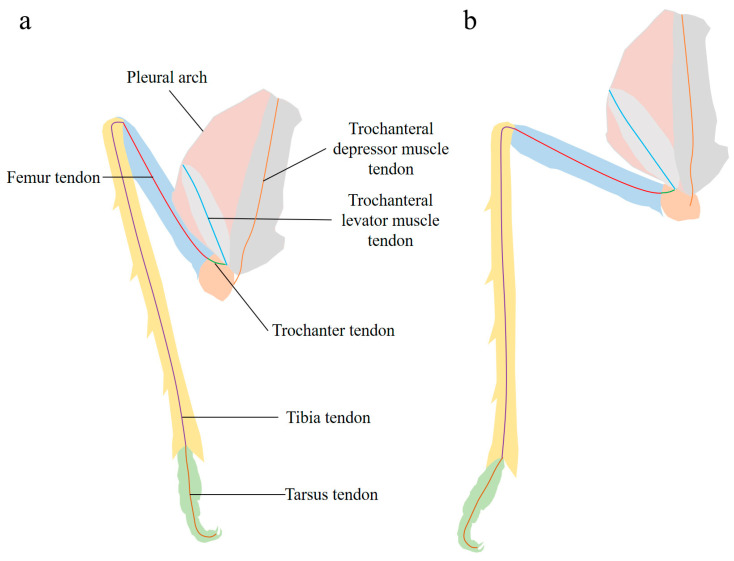
Schematic diagrams of the posture of each hind leg segment and tendons in the jumping structures. (**a**) Before jumping; (**b**) after jumping.

Tendons run through the cavities of the femur, tibia, and tarsus (Figure 8). These tendons are flexible components, contrasting with the rigid exoskeleton. During the preparation for jumping, the tendon connecting the trochanter and femur is tensioned by the turning of the trochanter and the elevation of the femur. Simultaneously, the tendons in the femur, tibia, and tarsus also become tensioned, maintaining each segment taut and folded (Figure 8a). When jumping, the taut and folded segments unfold rapidly (Figure 8b), leveraging the body forward. Based on these structures and their movements, we created a simplified model of this component (Figure S1) and simulated the entire process in a supplementary material video (Supplementary Material Video S2). The simplified model of the hind leg and tendon in Figure S1 represents the key structural elements of the lever component. It shows the arrangement of the femur, the tibia, the tarsus, and the associated tendons. This model simplifies the complex biological structure, making it easier to understand the basic principles of how the lever component functions during the jumping process. Video S2 presents a simulation of the hind leg movements during the jumping action. It animates the changes in the angles of different leg segments, such as the femur, tibia, and tarsus, as well as the role of tendons in tensioning and releasing energy.

### 3.2. Kinetics

**Figure 9:**
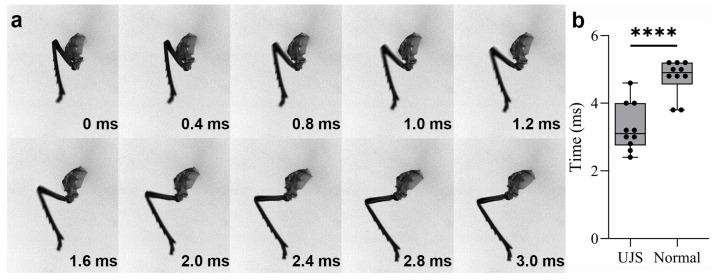
The jumping-like motion duration observed in *L. delicatula* while the unilateral jumping structures (UJSs) were manually moved. (**a**) The process of the jumping-like motion of the UJS. Images at crucial time points from a high-speed video, which was captured at a rate of 5000 frames per second. (**b**) The duration of the jumping-like motion and normal jumping (N = 10). **** indicates significant difference (*p* < 0.0001) based on the two-tailed unpaired *t*-test.

The leg movement was triggered by the rotating of the trochanter, which was 0.2–0.4 ms (1–2 frames) earlier than the deforming of the pleural arch (Figure 9a) (supplemental Video S3). Video S3 offers a detailed and slow-motion view of the leg movement of the UJS. It enables us to precisely track the key events of the leg movement, including the rotation of the trochanter, the deformation of the pleural arch, and the temporal changes in angles, which are crucial for understanding the kinetics of this jumping-like motion. The completion times of a jumping-like motion of the UJS and normal jump ranged from 2.4 ms to 4.6 ms and 3.8 ms to 5.2 ms (N = 10), respectively; the duration of the jumping-like motion of the UJS was significantly shorter than that of a normal jump (*p* < 0.0001, t = 5.322, df = 18) (Figure 9b).

**Figure 10:**
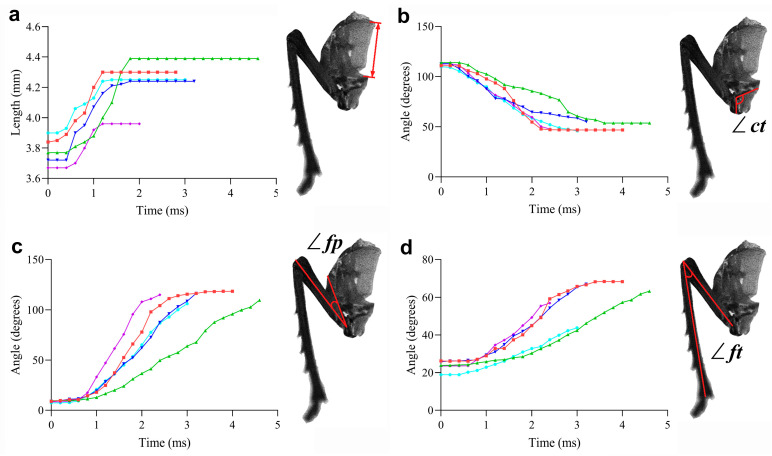
The time variation of (**a**) the length of the pleural arch, (**b**) the angle between the coxa and the trochanter (∠ct), (**c**) the angle between the femur and the pleural arch (∠fp), and (**d**) the angle between the femur and the tibia (∠ft). Five typical jumping-like motions observed while manually moving the UJS were selected to illustrate these variations, and lines of the same color denote the same jump. The insets in each figure are employed to show the measuring approaches of the variables.

Before jumping, the pleural arch length of the UJSs and individual insect were 3.72 ± 0.07 mm and 3.62 ± 0.11 mm (N = 10). After jumping, this length increased to 4.68 ± 0.06 mm and 4.64 ± 0.11 mm (N = 10). The calculated deformation lengths were 0.96 ± 0.06 mm and 1.02 ± 0.07 mm (N = 10) (Table 1). There is no significant difference in the length of the pleural arch between the UJS and individual insects. Additionally, the deformation time ranged between 0.8 and 1.4 ms (Figure 10a).

The angle between the coxa and trochanter of the UJS and individual insect before jumping (∠ct) were 111.4 ± 1.14 degrees and 110.90 ± 1.07 degrees (N = 10) (Table 1). The hind legs adopt a cocked position due to the tensile force exerted by the tendon. Once the jumping-like motion begins, the levator muscles relax, leading to the disassembly of the coxa–trochanter assembly. Consequently, the pleural arch reverses its direction, and the accumulated energy is instantaneously released. This prompts the trochanter to rotate due to the tensile force applied by the primary muscles. The ∠ct of the UJS and individual insect after jumping were 54.02 ± 1.01 degrees and 54.08 ± 0.95 degrees (N = 10) (Table 1). The period during which the angle between the coxa and trochanter undergoes significant changes spans from 0.4 milliseconds to 2.0 ms (Figure 10b).

Before jumping, the angle between the femur and pleural arch (∠fp) of the UJS and individual insect were 9.25 ± 0.36 degrees and 9.23 ± 0.35 degrees (N = 10). Following the jumping, this angle increases to 110.70 ± 1.52 degrees and 110.80 ± 1.41 degrees (N = 10) (Table 1). The interval during which the ∠fp undergoes substantial changes spans from 1.0 ms to 2.6 ms (Figure 10c).

Before jumping, the angle between the femur and tibia (∠ft) of the UJS and individual insect were 23.24 ± 0.90 degrees and 23.32 ± 0.79 degrees (N = 10). Once the jumping was executed, this angle of the individual insect (107.40 ± 0.98 degrees (N = 10)) was significantly bigger than that of the UJS (59.31 ± 2.55 degrees (N = 10)) (Table 1). The duration during which the ∠ft undergoes substantial changes is from 1.4 ms to 3.0 ms (Figure 10d).

## 4. Discussion

In this study, the anatomy of jump structures of adult *L. delicatula* was vividly depicted. More significantly, based on our discovery that the unilateral jumping structures of *L. delicatula* could function independently even when detached from the body (supplementary material Video S1), we endeavored to identify the key structures involved and elucidate how these components collaborate as a relatively autonomous and functional unit. This unit comprises a class of elastic mechanisms, where the storage and release of energy are mediated by latch-like opposing forces. This unit closely resembles the sophisticated mechanisms found in the legs of froghoppers [26], the legs of grasshoppers [38], the formidable raptorial appendage of mantis shrimps [24,39], and even the remarkable jaws of trap-jaw ants [23], highlighting its evolutionary convergence and functional elegance.

The features of UJS studied in this study are likely to underpin jumping mechanisms in many Hemiptera and beyond, offering a template for both biological inquiry and engineering innovation. Future comparative studies across taxa (e.g., cicadas, treehoppers) will clarify the universality of these findings and refine their applications in biomimetics. The Hemiptera order is highly diverse, encompassing a wide range of species with varying morphological and behavioral characteristics. However, many species within Hemiptera share similar jumping mechanisms [2,9,16], suggesting that the UJS’s structure and function may be conserved across different species. This conservation of jumping mechanisms across different species within Hemiptera indicates that the findings of this study could be applicable to a broader range of species.

The relative independence of each side’s jumping structures could allow them to operate without significant interference, potentially enhancing the efficiency and response speed of jumping. The UJS exhibited a jumping-like motion akin to that occurring within a living organism. Prior to the jump, no statistically significant differences were observed between the UJS and individual insects in terms of pleural arch length, ∠ct, ∠fp, and ∠ft. Post-jump, aside from ∠ft, the variations in other angles and pleural arch length remained comparable between the UJS and the insects. However, they are not completely separated and independent from each other. The jumping behavior of live insects is regulated by the nervous system [10,13,40]. While the Unilateral Jumping System (UJS) operates autonomously in vitro, its in vivo functionality depends on neural coordination [12,40]. In vitro, the UJS’s jumping-like motion is likely initiated by muscle tension disrupting the latch state between the coxa and trochanter, and this is a process driven by passive mechanical forces. Conversely, in vivo, muscle activation is neurologically controlled by motor neurons, transforming this mechanism into an active, neural-driven process. Simultaneously, neural and mechanical mechanisms ensure the synchronization of movements and energy storage between both sides during jumping [7,13]. The synchronization mechanisms make both sides connected and move in coordination when jumping [7]. During its jumping-like motion, the UJS demonstrated a shorter duration compared to normal jumping. This discrepancy may likely be attributed to the combined effects of the insect’s body mass and its neural control mechanisms. These findings suggest that while synchronization of both hind legs is crucial for insect jumping, each side of the jumping structures possesses its own independence and functional integrity.

In this study, the UJS was analyzed as an isolated functional unit. However, it is an integral component of the insect’s anatomical system. The dynamic interactions between the UJS and other body structures—such as the wings, the other legs, and the thoracic musculature—likely modulate jumping dynamics and stability. Consequently, the current experimental framework cannot replicate key biological phenomena, including aerodynamic effects or multi-limb coordination (e.g., the stabilizing role of wings during take-off or the synergistic engagement of flight muscles in intact insects). These limitations underscore the necessity of integrating systemic interactions in future models to fully capture the complexity of natural jumping behavior. Future research should explore these interactions and comparisons to better understand the integration of the UJS within the insect’s overall locomotor system. Additionally, minor pleural arch structures (e.g., protuberances) warrant further study to fully model deformation mechanics. The high-speed recordings and anatomical dissections provided detailed insights into the UJS’s mechanics. However, these methods may not capture all aspects of the jumping mechanism, such as the exact forces involved in the energy storage and release processes. Future studies could incorporate additional techniques, such as force measurements or more advanced imaging methods, to provide a more comprehensive understanding.

**Figure 11:**
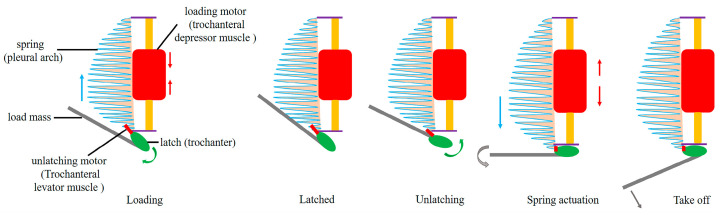
The schematic illustration depicts the jumping action mechanism of the UJS in *L. delicatula*. It was crafted with reference to the work of Cook et al. [42], integrating relevant concepts and findings from their research to accurately represent the complex biomechanical processes involved in the jumping mechanism. This schematic aims to visually convey the key elements and interactions within the UJS during the jumping action, providing a clear and concise overview for readers to better understand the underlying principles.

The integration of the elastic and latch mechanisms in the UJS of *L. delicatula* is a key feature of its jumping ability. The pleural arch deformation and the trochanter’s rotation work together to store and release energy efficiently. According to this study and previous studies about the mechanisms of similar biological latch-mediated spring actuated systems [41,42], the UJS’s jumping action can be summarized to four stages: the loading, latched, unlatching, spring actuation, and take-off (Figure 11). Among the structures involved, the pleural arch acts as a sturdy component (spring), while the trochanteral depressor muscle serves as an actuator (loading motor). These structures are responsible for energy storage and release, making them energy storage modules. In addition to its stiffness, the pleural arch must possess moderate elasticity to prevent fractures and facilitate the body’s return to its original shape following a jump [7]. Similar to other insects that employ catapult jumping mechanisms, the trochanteral depressor muscle powers the jump by contracting to bend the pleural arch prior to jumping (loading state) [7,8,9,12,20,34,43]. When the contraction is complete, the internal core muscle is responsible for securing the bent pleural arch until the jump is initiated. Besides the trochanteral depressor muscle, there are several other small muscles that play auxiliary roles in jumping [16,21,22]. We found that the levator muscles (unlatching motor) play a crucial role in elevating the femur and tensioning the hind leg. The coxa and trochanter (latch) are integral components for the deformation of the pleural arch, as their angles and positions undergo modifications, allowing for energy to be accumulated gradually and released instantaneously.

The pleural arch comprises several distinct cuticular components with intricate shapes. In addition to the U-notch and V-notch mentioned in this study, there are numerous other minor structures (e.g., protuberances and indentations) on the pleural arch that might also contribute to its deformation. Therefore, further investigations are needed to fully understand the structure of the pleural arch and the associated mechanical processes involved in its deformation. The energy storage structures (i.e., pleural arch and muscles) and leverage structures (i.e., femur, tibia, and tarsus) are interconnected by the coxa and trochanter, which interact through their pivot connection. The pivot plays a pivotal role in the bending and recovery of the pleural arch, facilitating energy storage and release [6,16]. When the isolated jumping structures were artificially placed in an energy-storage state, the pivot remained in a stranded state (latched state). This state can be sustained for a period and ultimately broken apart by the strength of the trochanteral depressor muscle (unlatching state) (supplementary material Video S1).

In addition to the coxa and trochanter, the hind leg’s other components play a crucial role in converting stored energy into kinetic energy through leverage, propelling the insect into a jump (spring actuation and take-off) [44]. When a hind leg is fully raised and poised for a jump, the coxal protrusion interfaces with the white patch on the femur. The protrusion and patch of planthoppers are less complex compared to those of froghoppers [2]. In froghoppers, the two protrusions remain engaged, preventing the leg from unfurling during the prolonged contraction of the trochanteral depressor muscles [2,22,26]. Conversely, the coxal protrusion and patch of *L. delicatula* do not maintain strict engagement in readiness for jumping, allowing the femur to move within a range. This feature makes it more flexible when walking and climbing. These two structures only engage strictly just before take-off. We believe that the function of coxal protraction is to sustain the femur during jumping. Due to an altitude difference at the junction between the metathorax and abdomen (where the femur is located), this sustaining action prevents the femur from rocking up and down. Furthermore, the coxal protrusions of both sides likely play a critical role in keeping the femora of both sides in the same plane beneath the body. As a result, the hind legs are held under the body and moved in the same plane that is almost parallel to the longitudinal axis of the body [2]. In addition, their engagement may also provide information about the cocked position of a hind leg. The sharp spines on tibia and tarsus can provide traction during the acceleration phase by piercing substrates [45].

The deformation length of the pleural arch is a critical parameter that directly influences the energy storage capacity of the UJS. A longer deformation length allows for greater energy storage, which can result in more powerful jumps. However, there is a trade-off between deformation length and the structural integrity of the pleural arch. Excessive deformation could lead to fractures or reduced efficiency in energy release. In many jumping insects, such as planthoppers and froghoppers, the pleural arch is a composite structure comprising chitinous cuticle and resilin. Chitinous cuticle has been regarded as an energy storage mechanism primarily under limited deformation, whereas resilin is utilized for larger reversible deformations [5]. Resilin is likely to play a critical role in energy storage and release mechanisms of the pleural arch in *L. delicatula.* Hence, biomimetic devices should aim to maximize deformation length while maintaining structural integrity. This can be achieved by using materials with high tensile strength and elasticity, like the resilient properties of the pleural arch in *L. delicatula*.

The angles between the coxa and trochanter (∠ct), femur and pleural arch (∠fp), and femur and tibia (∠ft) undergo significant changes during the jumping process. These changes are crucial for the efficient transfer of energy from the storage phase to the release phase. The reduction in ∠ct during the jump indicates a rapid release of stored energy. This angle change is essential for the backward rotation of the coxa, which propels the insect forward. The increase in ∠fp suggests a significant change in the position of the femur relative to the pleural arch, facilitating the unfolding of the leg segments and the conversion of stored energy into kinetic energy. The increase in ∠ft indicates the extension of the tibia, which is crucial for generating the force needed for the jump. The detailed analysis of the UJS components and their motion changes has revealed significant insights into the overall performance of the jumping mechanism in *L. delicatula*. Variations in the angles (∠ct, ∠fp, and ∠ft) and the deformation length of the pleural arch directly influence the efficiency and speed of the jumping action. Understanding these relationships can provide valuable guidance for biomimetic design. The integration of elastic energy storage and precise mechanical latching mechanisms, as observed in the UJS, can inspire the development of more efficient and robust biomimetic jumping devices. By mimicking the seamless integration of diverse structures within a single unit, engineers can design systems that enhance performance while minimizing complexity. This approach not only improves the functionality of biomimetic mechanisms but also opens new avenues for innovation in fields such as robotics and mechanical engineering.

The exoskeletons of various insects are equipped with specialized structures that fulfill specific functions. These structures can be categorized into distinct functional zones [46]. In the present study, we abstracted the jumping structures of *L. delicatula* and identified the key components. This study demonstrates the functional autonomy of the UJS, driven by integrated energy storage, coupling, and lever systems. By forging a connection between biomechanics and engineering, the findings of this study present a valuable template for the development of efficient, modular, and rapid energy-storage-release mechanisms. The UJS’s ability to store and release energy rapidly can inspire the design of bionic insect flight robots, especially during the take-off phase.

The integration of diverse jumping structures in the *L. delicatula* is a fascinating example of how insects have evolved to optimize functionality through the combination of different anatomical features. This approach reveals a widespread strategy that is employed by insects to create efficient and effective mechanisms for locomotion and escape. This integration is not just a random combination of parts but rather a highly evolved and specialized adaptation that has been optimized over millions of years of evolutionary history. By studying and understanding these integrated systems, we can gain insights into how nature has optimized functionality and performance through evolution. This knowledge may also inspire new design principles and solutions in engineering and technology.

## Figures and Tables

**Table 1:** The change in the pleural arch length and leg angles before and after jumping.

Structures	Biological Significance	UJS/Normal Jump	Before Jumping	After Jumping	Variation
			*N* = 10 (Mean ± SE) *		
Pleural arch length (mm)	The deformation length is crucial for energy storage and release during the jumping-like motion.	UJS	3.72 ± 0.07	4.68 ± 0.06	0.96 ± 0.06
		Normal	3.62 ± 0.11	4.64 ± 0.11	1.02 ± 0.07
Angle between coxa and trochanter (∠ct) (deg.)	This angle reflects the positioning and movement of the coxa and trochanter, which are key components in the coupling mechanism of the UJS.	UJS	111.40 ± 1.14	54.02 ± 1.01	57.42 ± 1.60
		Normal	110.90 ± 1.07	54.08 ± 0.95	56.78 ± 1.32
Angle between femur and pleural arch (∠fp) (deg.)	This angle indicates the bending and recovery of the pleural arch, which is essential for energy storage and release.	UJS	9.25 ± 0.36	110.70 ± 1.52	101.40 ± 1.59
		Normal	9.23 ± 0.35	110.80 ± 1.41	101.60 ± 1.54
Angle between femur and tibia (∠ft) (deg.)	This angle represents the movement of the femur and tibia, which are critical for converting stored energy into kinetic energy during the jumping-like motion.	UJS	23.24 ± 0.90	59.31 ± 2.55 ^a^	36.06 ± 2.41 ^a^
		Normal	23.32 ± 0.79	107.40 ± 0.98 ^b^	84.03 ± 0.83 ^b^

Notes 1: * The length marked with different superscript letters in the same column, indicate significant differences (*p* < 0.001) between the UJS and normal jumping, as determined by a two-tailed unpaired *t*-test. Notes 2: UJSs, unilateral jumping structures; normal, normal jump of female; variation, the absolute differences in lengths and angles between before jumping and after jumping.

## Data Availability

Data are contained within the article.

## Supplementary Materials

The following supporting information can be downloaded at https://www.mdpi.com/article/10.3390/biomimetics10070444/s1, Figure S1: A simplified model of the hind leg and tendon in it; Video S1: The excised unilateral jumping structures of *L. delicatula* and the jumping action; Video S2: The simulated movements of the hind leg in jumping action; Video S3: The jumping action of the unilateral jumping structures of *L. delicatula* taken at 5000 frames per second.

## Appendix A. Glossary of Technical Terms in Jumping Structures of *L. delicatula*

**Jargon**	**Definition**
Unilateral jumping structures (UJS)	A functional structural unit of *L. delicatula* that can relatively independently perform jumping actions. It consists of the pleural arch, coxa, and hind leg and can be divided into an energy storage component, a coupling component, and a lever component.
Pleural arch	A part of the internal skeleton of the thorax of *L. delicatula*, which is bow-shaped, located on both sides of the metathorax, and fused with the coxa. It deforms while jumping to store and release energy.
Trochanteral depressor muscles	The muscles mainly consist of peripheral muscle fibers and a core part. They contract to bend the pleural arch for energy storage, being crucial muscles for the storage and release of energy in jumping.
Coxa	One segment at the base of the hind leg, which is closely connected to the pleural arch. It is part of the coupling component and plays a role in connecting and transmitting forces in the jumping mechanism.
Trochanter	Located between the coxa and the femur, it interacts with the coxa through protuberances and pivots. Its structure and movement affect the realization of jumping actions.
Femur	A part of the hind leg and an important component of the lever component. During the preparation and execution of jumping, it collaborates with other leg structures to help the insect complete the jumping movement.
Tibia	There are four spines on the hind leg. The ventral side of its distal margin has sclerotized spines. It participates in leg movement through extension and contraction during jumping, playing an important role in the propulsion and stability of jumping.
Tarsus	It consists of two tarsal segments and a pretarsus. The pretarsus includes a pair of claws and an arolium. It is used to stabilize the body and prevent slipping during jumping.
V-notch, U-notch	Structures on the pleural arch. The deformation zone extends from the V-notch to the U-notch, accounting for approximately two-thirds of the pleural arch and playing an important role in the deformation process of the pleural arch.
∠ct	The angle between the coxa and the trochanter. It changes significantly during the preparation and execution of jumping, reflecting the movement state of the leg joint and the mechanical changes of the jumping mechanism.
∠fp	The angle between the femur and the pleural arch. The angle changes obviously before and after jumping, which is an important indicator to measure the relative movement between the leg and the energy- storing structure during the jumping process.
∠ft	The angle between the femur and the tibia. The angle increases during jumping, and its change has an important impact on the leg extension and the generation of jumping propulsion force.
