# Supplementary material for: The Unilateral Jumping Structures of the Spotted Lanternfly, Lycorma delicatula (Hemiptera: Fulgoridae): A Highly Functional and Integrated Unit
Source: Biomimetics (Basel). 2025 Jul 6;10(7):444. doi: 10.3390/biomimetics10070444 (PMC12293068; doi:10.3390/biomimetics10070444)
Supplement: Supplementary file 1 [file biomimetics-10-00444-s001.zip › Supplementary material.pdf]

The unilateral jumping structures of the spotted lanternfly, *Lycorma delicatula* (White) (Hemiptera: Fulgoridae): a highly functional and integrated unit

Supplementary figure:

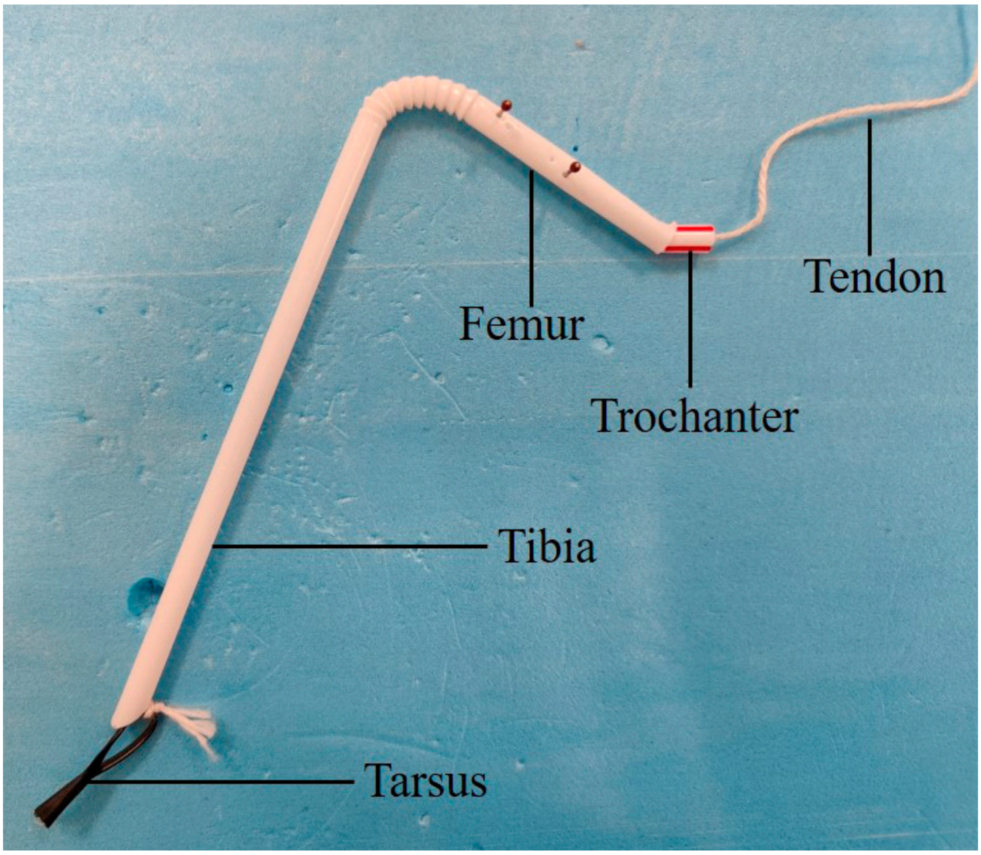

Figure S1. A simplified model of hind leg and tendon in it.

Supplementary movies:

Video S1. The excised unilateral jumping unit of *L. delicatula* and the jumping action.

Video S2. The simulated movements of hind leg in jumping action.

Video S3. The jumping action of unilateral jumping unit of *L. delicatula* taken at 5000 frames per second.
